# Colostrum feeding practice and associated factors among mothers who come for postnatal care to Asella referral and teaching hospital, Arsi Zone, South-East Ethiopia

**DOI:** 10.3389/fmed.2024.1487179

**Published:** 2025-01-07

**Authors:** Gisha Haji, Gebi Agero, Amde Eshete, Abdurahman Hasan, Ahmed Hiko

**Affiliations:** ^1^School of Public Health, College of Health Sciences, Arsi University, Asella, Ethiopia; ^2^School of Nursing and Midwifery, College of health sciences, Arsi university, Asella, Ethiopia; ^3^School of Nursing and Midwifery, College of Health and Medical sciences, Haramaya university, Harar, Ethiopia

**Keywords:** colostrum feeding, practice, post-Natal, Asella, Southeast Ethiopia

## Abstract

**Background:**

Every day throughout the world more than 4,000 infants and young children die because colostrum feeding was not initiated within an hour of birth as recommended by the World Health Organization (WHO)/United Nations Children’s Fund. Even though breastfeeding is common in Ethiopia, the widespread belief that colostrum feeding causes morbidity and mortality among neonates still exists.

**Objective:**

To assess the colostrum feeding practice and It’s associated factors among mothers who come for Post-natal care at Asella Referral and Teaching Hospital, Asella Town, Southeast Ethiopia from November 20, 2023, to January 25, 2024.

**Method:**

This study employed an institutional-based cross-sectional study among 301 surveyed postnatal care mothers from November 20, 2023, to January 25, 2024. Systematic random sampling was employed to select study participants. A pretest was performed to check the reliability and clarity of the study questionnaires. The tool to examine the practice of colostrum feeding and related aspects was modified from earlier studies. The collected data were input into Epi Info version 7.2.5.0 and exported to SPSS version 26 for analysis. Bivariate and multivariable logistic regression analyses were used to identify variables associated with the practice of colostrum feeding. The variables with significant association were identified based on *p*-value < 0.05.

**Results:**

In this study, 301 study participants took part, with a 99% response rate. The mean age of mothers was found to be 27.13 ± 5.46. This study’s overall prevalence of practice was 57.8% (95% CI, 52.2–63.35). Being Urban residence (AOR = 4.05, 95%CI: 2.18–7.52), health education counseling (AOR = 4.31, 95%CI: 1.27–10.73), and favorable attitude toward colostrum milk (AOR = 6.64, 95% CI: 3.61–12.2) were factors significantly associated with colostrum feeding practice.

**Conclusion and recommendations:**

In comparison to WHO recommendations, the study area had a low rate of colostrum feeding practice. Additionally, this study also identified factors associated with maternal colostrum feeding practice level such as urban residence, counseling on timely initiation of breastfeeding during antenatal care, and good maternal attitude toward colostrum feeding. Therefore, Health education dissemination should be given to postnatal mothers regarding the importance of colostrum feeding.

## Introduction

Colostrum is the thick, yellowish fluid secreted by the breast beginning in midpregnancy and continuing for 3–4 days after delivery. It has a high concentration of antibodies and other protective elements essential for newborns (1). It contains antibodies which help the baby to fight against illness. Furthermore, early breastfeeding helps to forge the bond between mother and baby as well as enable a constant milk supply (2). Compared to regular milk, colostrum has much lower levels of lipids and potassium, but it is rich in proteins, carbohydrates, vitamin A, and sodium chloride. Its laxative properties, along with bioactive immune components, help protect newborns from various illnesses and allergic conditions (3). Besides being a pure food source, breast milk provides all the essential nutrients a baby needs during the first 6 months of life. The American Academy of Pediatrics recommends exclusive breastfeeding for the first 6 months (2).

The two main components of colostrum are also growth factors and immune factors. Colostrum nursing allows the baby to receive the mother’s passive immunity, which aids in the infant’s defense against bacteria, viruses, fungi, giardia, and other protozoan parasites. This comprehensive defense is something even antibiotics cannot offer. Moreover, unlike synthetic antibiotics, colostrum does not lead to bacterial resistance. As a result, it supports the infant’s healthy development, strength, and longevity (4, 5). Colostrum is also a great source of vital growth factors, which are important for promoting growth, promoting regeneration, and accelerating the repair of aging skin, muscles, collagen, bone cartilage, and nerve tissue (6). In addition, it contains high levels of sodium, chloride, and cholesterol, which collectively support the optimal development of the infant’s heart, brain, and central nervous system (7, 8).

Globally, less than half of newborns begin breastfeeding within the first hour of life. As a result of not receiving colostrum within this crucial time frame, over 4,000 infants die worldwide each day (9). In 2015, approximately 2.7 million newborns died globally within the first month of life (0–27 days), with more than a third of these deaths occurring on the first days (10). Southeast Asia and Sub-Saharan Africa together account for two-thirds of the nearly 3 million newborn deaths that occur annually (1). Sub-Saharan Africa continues to have the highest infant mortality rates in the world. In developing countries, 3,000–4,000 newborns die daily from acute respiratory diseases and diarrhea because they do not receive adequate breast milk and colostrum (11). According to the World Health Organization (WHO), neonatal deaths contribute to 45% of all deaths in children under the age of five (12). While three-quarters of newborn deaths happen within the first 7 days of life, over one-third of these deaths occur within the first 24 h after birth (13).

Numerous studies suggest that children who did not receive colostrum are at a higher risk of wasting, infections, stunting, and underweight (14–16). Avoiding colostrum also reduces a newborn’s intake of immunoglobulin and other vital nutrients, which impairs the gastrointestinal tract’s priming and raises the risk of infant morbidity and mortality (17). Despite the long-term health and immunological benefits that colostrum offers, it is often avoided in many countries, including Nepal, Pakistan, and Burkina Faso, where a significant number of mothers discard colostrum (18–20). In Ethiopia, discarding colostrum is a widespread nutritional malpractice across nearly all communities. For instance, in Afar, 35% of mothers reported discarding colostrum, in Debra Tabor, the rate was 25.6%, in Raya Kobo it was 13.5%, in Axum it was 6.3%, in Jinka Town it was 9.8%, and in Motta Town it was 20.3% (21–24).

In developing countries, among women who do not give colostrum feeds to their children, most of them avoid colostrum feeding based on traditional or cultural beliefs (14, 25, 26). Ethiopia has one of the highest infant mortality rates in the world that occur due to inappropriate neonatal feeding. Even though colostrum discarding hurts child health, little is known about the extent of the problem and its contributing factors in South Eastern Ethiopia (11). Furthermore, in Ethiopia, a substantial number of neonatal deaths occur within the first week of life, and many mothers hold the misconception that colostrum feeding is linked to neonatal morbidity and mortality. As a result, they believe that colostrum should be discarded. However, the reasons behind this misconception have not been thoroughly studied in Ethiopia, particularly in the study area. Therefore, this study aimed to assess the Colostrum feeding practice and identify its associated factors among mothers attending postnatal care at Asella Referral and Teaching Hospital, located in Asella Town in Southeast Ethiopia.

## Objectives

### General objective

To assess colostrum feeding practice and its associated factors among mothers attending post-natal care service in Asella Referral and Teaching Hospital from November 20, 2023, to January 25, 2024.

### Specific objectives

To determine the colostrum feeding practice among mothers attending post-natal care service in Asella Referral and Teaching Hospital from November 20, 2023, to January 25, 2024.

To identify factors associated with colostrum feeding practice among mothers attending post-natal care service in Asella Referral and Teaching Hospital from November 20, 2023, to January 25, 2024.

### Specific objectives

**Figure 1:**
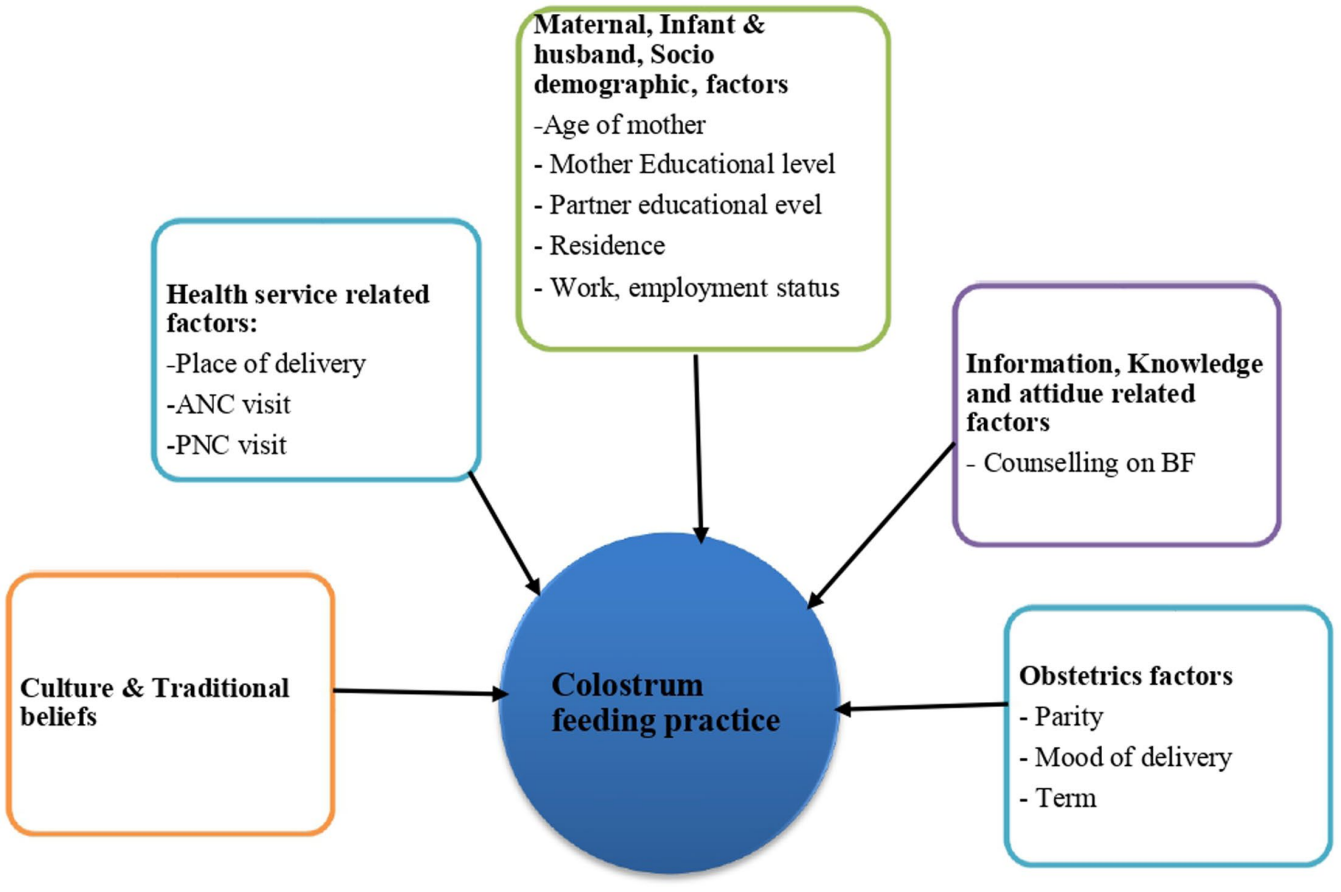
Theoretical framework for the colostrum feeding practice and its associated factors among Mothers Who Come for Postnatal Care to Asella Referral and Teaching Hospital, Arsi Zone, South-East Ethiopia, 2024.

After reviewing different literature, the following theoretical framework is developed as indicated in Figure 1.

## Materials and methods

### Study design and setting

**Figure 2:**
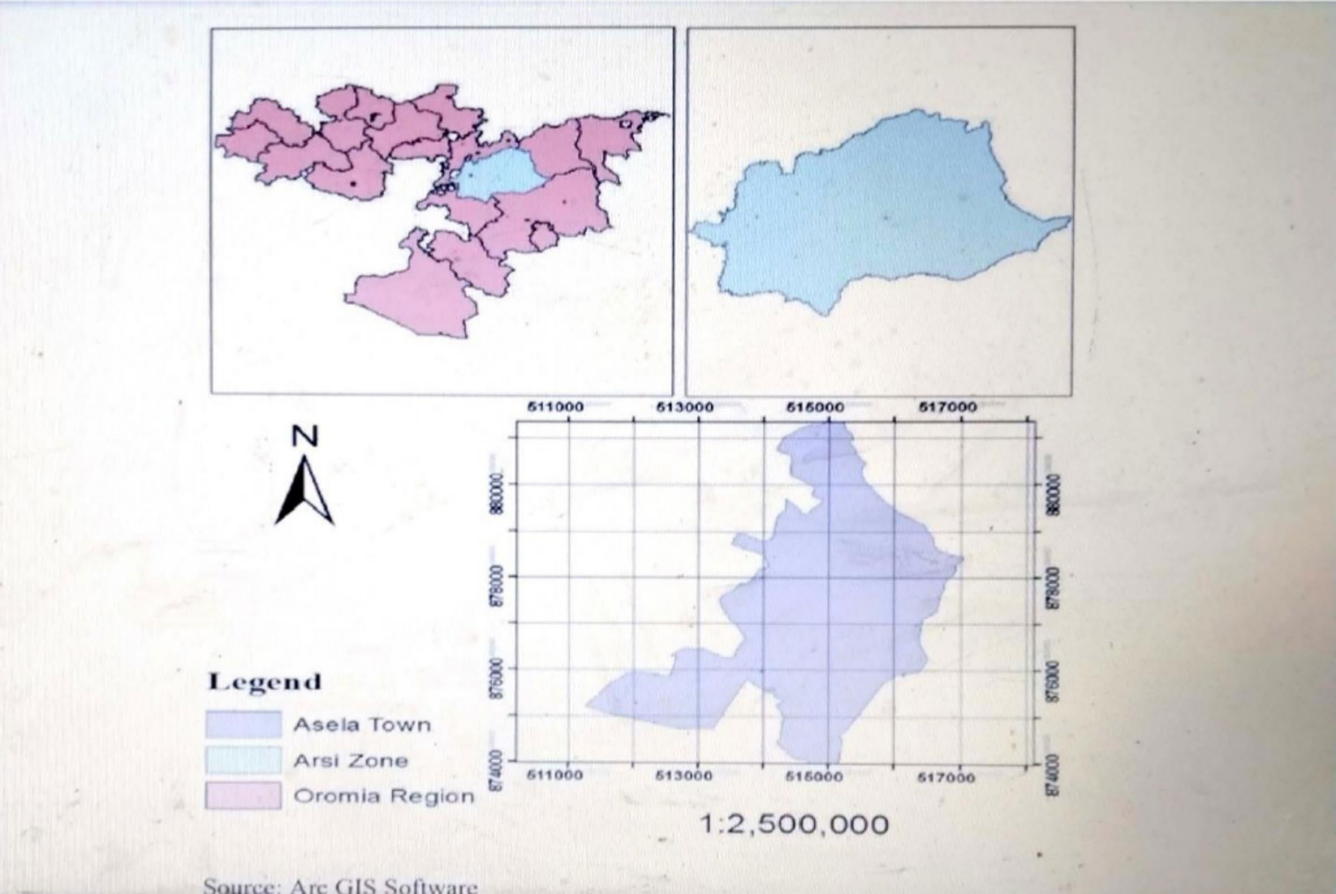
Map of Ethiopia showing the location of study area Asella Towns, 2024.

This study was conducted from November 20, 2023, to January 25, 2024, at Asella Referral and Teaching Hospital, located in Arsi Zone, Oromia Regional State (Figure 2). The hospital was established in 1964 and is situated about 175 kilometers southeast of the capital city, Addis Ababa. Currently, the zone has a total population of around 3,563,474 of which 1,767,483 were females. Of the total number of women, approximately 44.8% were in the reproductive age group. According to the hospital’s annual report provided by the administration, it has a total of 443 staff members. Asella Referral and Teaching Hospital offers preventive, curative, and diagnostic services to the population of approximately 3.5 million in Asella town and its surrounding areas. The hospital operates through three main departments: outpatient department (OPD), inpatient department, and emergency department. There are several services available for mothers and newborns at the hospital, such as antenatal care (including investigations and immunizations), delivery services, postnatal care (PNC) services, and an expanded program on immunization (EPI) services. According to the hospital’s quarterly report, around 1,254 mothers got care there. From these, 289 were from the obstetrics and gynecology follow-up department, 553 from the obstetric ward, and 412 from the EPI department.

### Source and study populations

The source populations were all mothers who had children less than 6 months of age and who attended postnatal care at Asella Referral and Teaching Hospital. Mothers who had children less than 6 months of age, Attending Asella Referral and Teaching Hospital, and were eligible for the study were included in the study. However, mothers who had severe mental illnesses or were unable to participate in the study during data collection were excluded from the study.

### Sample size determination

The required sample size was determined by a formula with a single population. The calculation was made assuming 95% confidence interval, 5% margin of error and the prevalence of 56.5% colostrum feeding practice reported in a study done in Ambo (26). In this study, the calculation for correctional formula was applied because the sample from which population drawn is less than 10,000 and by adding non-response rate of 10%, sample size final became 304. For the second objective (Factors associated with colostrum feeding practice), we used double population proportion formula in Epi info version 7 statistical calculation to determine a sample size as indicated in Table 1.

**Table 1:** Sample size determination for the study on the colostrum feeding practice and associated factors among mothers who come for Postnatal Care to Asella Referral and Teaching Hospital, Arsi Zone, South-East Ethiopia.

Variables	Assumptions	% outcome in unexposed	% outcome in exposed	Calculated sample size with 10 non-response rate	References
Delivery place	CI: 95% Power: 80 Ratio: 1:1	31.4	71.6	61	(37)
Counseling	CI: 95% Power: 80 Ratio: 1:1	24.8	53.6	112	(37)
Knowledge	CI: 95% Power: 80 Ratio: 1:1	29.8	84.1	35	(37)

Therefore, we selected the higher result by comparing the result of two of the objectives and the final sample was 304.

### Sampling procedure

To select the study participants, the quarterly report’s total number of mothers enrolled in the healthcare facility, 1,254 was determined. Once the *K* value had been calculated, respondents were selected by a systematic random sampling method. The first participant was randomly selected and further participants were recruited every four.

### Data collection tools and methods

The questionnaires were developed by reviewing previous studies (1, 3, 8, 17, 26). Initially, the questionnaire was prepared in English, then translated into the local language, Afan Oromo, to facilitate data collection from the study participants. Finally, it was translated back into English by experts to ensure consistency. The tool comprised four sections: socio-demographic factors, knowledge-related factors, attitude-related factors, and practice-related factors. Data was collected by three BSc Nurses, using a structured face-to-face, interviewer-administered questionnaire and supervised by one MSc nurse.

### Study variables and measurements

In this study, the outcome variable was colostrum feeding practice and the independent variables were socio-demographic characteristics, maternal health service utilization, knowledge of mothers about colostrum, and attitude of mothers toward colostrum. Colostrum is the breast milk women produce in the first few (3, 4) days after birth before the regular breast milk comes (17). Colostrum feeding practice is defined as the mothers who give colostrum (first thick and yellowish milk) to the newborn within the first 3 days of birth (26). Feeding colostrum was coded as “1” while avoiding colostrum feeding was coded as “0” for regression analysis.

Good knowledge among participants was defined as having responses equal to or greater than the mean value of the total knowledge-related questions, while those who scored below the mean were categorized as having poor knowledge (3). Similarly, a good attitude was determined by responses equal to or above the mean value for attitude-related questions, with responses below the mean coded as a poor attitude (3). Moreover, if the mother responded greater than or equal to the mean value to practice-related questions, it is coded as good practice, while if the mother responded less than the mean value, it is considered poor practice (3).

### Data quality controls

To minimize bias, data collectors were recruited from outside the study area. The principal investigator provided 1 day of training to the data collectors and supervisors, covering the study’s objectives, components, participant recruitment, ethical considerations, and a general overview of the data collection procedure to ensure a common understanding. To enhance data quality, a pretest was conducted on 5% of the total sample outside the study setting prior to the actual data collection. Feedback from this pretest was incorporated into the survey. Every day, the supervisors rechecked the questionnaire’s completeness after data collection was completed.

### Data processing and analysis procedure

Data were entered into Epi Info version 7.2.5.0 and exported to SPSS version 26 for analysis. Descriptive analysis results were presented in the form of tables and texts using frequencies and summary statistics such as standard deviation and percentage. A binary Logistic regression model was also used to identify factors associated with the practice of colostrum feeding practice. Then those variables with a *p* value of < 0.25 were entered into multivariable logistic regressions to control the effect of confounding variables. An adjusted odds ratio with a 95% Confidence Interval (C.I) was calculated to determine the strength of the association. The model goodness of fit was tested by the Hosmer-Lemeshow and Omnibus test. Accordingly in this study, Hosmer-lemshow and Omnibus test (*p* = 0.47 and *p* = 0.000) respectively. To check the presence of multicollinearity among independent variables variance inflation factors >10 and tolerance <0.1 were used. Finally, to declare statistical significance, a *p*-value of < 0.05 was considered.

### Ethical considerations

To ensure ethical standards, necessary permissions were sought from Arsi University Asella Referral and Teaching Hospital, the Department of Public Health, as well as the administrative body of the hospital, to obtain cooperation for the study. The study, purpose, procedure, duration, possible risks, and benefits of participating in this study were clearly explained for each study participant using the local language (Afan Oromo). Verbal and informed consent was taken from each study participant before data collection and the right to withdraw was informed.

## Results

### Socio-demographic characteristics

In this study, a total of 301 study participants were surveyed, making a response rate of 99%.

The mean (±SD) age of study participants was 27.13 (±5.46) years. Of the total respondents, 189 (62.8%) aged from 25 to 34 years old and the majority of the respondents, 294 (97.7%) were married, 233 (77.4%) had formal education, and 159 (52.8%) of the respondents were Muslim religion followers. Of the total respondents, 208 (69.1%) of mothers were housewives, and 187 (62.1%) of mothers resided in rural areas (Table 2).

**Table 2:** Socio-demographic and economic-related characteristics of mothers who attended the PNC clinic at Asella Referral and Teaching Hospital, Southeast Ethiopia, 2024 (*n* = 301).

Characteristics	Category	Frequency	Percent
Age of mothers	18–24	69	22.9
	25–34	189	62.8
	≥35	43	14.3
Sex of neonate	Male	149	49.5
	Female	152	50.5
Maternal ethnicity	Amhara	69	22.9
	Oromo	212	70.4
	Silte, Gurage, and Tigray	20	6.6
Maternal religion	Orthodox	102	33.9
	Muslim	159	52.8
	Protestant	40	13.3
Residence	Urban	114	37.9
	Rural	187	62.1
Maternal marital status	Married	294	97.7
	Divorced, Windowed, and single	7	2.3
Maternal educational level	Informal education	68	22.6
	Formal education	233	77.4
Maternal occupation	Housewife	208	69.1
	Governmental employee	38	12.6
	Merchant	23	7.6
	Others (students, daily labor)	32	10.6
Father or husband’s educational status	Unable to read and write	14	4.7
	Read and write only	82	27.2
	Primary school (1–8)	67	22.3
	Secondary school (9–10)	92	30.6
	Collage and above	46	15.3
Under five	<2	154	51.2
	≥2	147	49.8
Household monthly income	<5,000	74	24.6
	5,000–10,000	160	53.2
	>10,000	67	22.3

### Obstetrics health service-related characteristics of mothers

This study showed that 283 (94%) of mothers, received antenatal care for their recent pregnancies. From these, 227 (80.2%) of these mothers attended ANC appointments four or more times, yet only 47 (16.6%) received health education regarding breastfeeding and colostrum. The majority of mothers chose to have deliveries at a health facility 294 (97.7%). Regarding the mode of delivery, 152 (50.5%) experienced vaginal delivery (Table 3).

**Table 3:** Obstetric health services related to characteristics of mothers who attended PNC clinic at Asella referral and teaching hospital, Oromia regional state, Southeast Ethiopia, 2024 (*n* = 301).

Characteristics	Category	Frequency	Percent
Place of birth	Home	7	2.3
	Health facilities	294	97.7
Parity	Primiparity	112	37.2
	Multiparty	189	62.8
ANC service for recent pregnancy	No	18	6.0
	Yes	283	94.0
How many times did you receive ANC (*N* = 283)	<4	56	19.8
	≥4	227	80.2
Did you receive health education on breastfeeding and colostrum (*N* = 283)	No	236	83.4
	Yes	47	16.6
A follow-up center (*N* = 283)	Health center	149	52.7
	Public hospital	82	28.9
	Private hospital or clinic	52	18.4
Mode of delivery	Vaginal delivery	152	50.5
	Instrumental delivery	43	14.3
	Cesarean section	106	35.2
Maternal known medical illness	Yes	39	13.0
	No	262	87.0

### Knowledge of colostrum feeding

Regarding knowledge about colostrum, 251 (83.4%) of mothers were generally aware of colostrum feeding. Additionally, 220 (73.1%) of the mothers recognized the color of colostrum. Of the total respondents, 195 (64.8%) of mothers were aware that colostrum milk serves as the first vaccine and 173 (57.5%) knew that initiating colostrum feeding immediately helps protect against maternal complications such as vaginal bleeding. In this study, the overall knowledge of mothers about colostrum feeding was 65.8% (95% CI, 60.46–71.19) (Table 4).

**Table 4:** Knowledge of colostrum feeding among mothers who attended PNC clinic at Asella referral and teaching hospital, Oromia regional state, southeast Ethiopia, 2024 (*n* = 301).

Characteristics	Category	Frequency	Percent
Did you know colostrum milk?	No	50	16.6
	Yes	251	83.4
Colostrum is thick, sticky, and yellowish	No	81	26.9
	Yes	220	73.1
If you know from where did you hear? (*N* = 251)	Health professional	146	58.2
	Mass media	29	11.6
	Family	57	22.7
	Friends and neighbor	19	7.6
Colostrum feeding is initiated within an hour and continues for 3 days after birth	No	72	23.9
	Yes	229	76.1
Colostrum is high in protein and helps the baby to grow	No	73	24.3
	Yes	228	75.7
Colostrum is the first vaccine given to the baby	No	106	35.2
	Yes	195	64.8
Colostrum protects disease from babies	No	76	25.2
	Yes	225	74.8
Colostrum milk protects vaginal bleeding	No	128	42.5
	Yes	173	57.5
Maternal Knowledge	Poor	103	34.2
	Good	198	65.8

### Attitude toward colostrum feeding

In this study, 181 (60.1%) of mothers agreed that colostrum is important for growth and mental development. About 70 (24.9%) of mothers believed that colostrum causes diarrhea and 69 (22.3%) thought that colostrum breast milk is dirty and looks like pus. The findings revealed that approximately 62.8% (95% CI: 57.36–68.23) of mothers had favorable attitudes toward colostrum feeding (Table 5).

**Table 5:** Attitude toward colostrum feeding among mothers who attended PNC clinic at Asella referral and teaching hospital, Oromia regional state, Southeast Ethiopia, 2024 (*n* = 301).

Characteristics	S. Agree	Agree	Neutral	Disagree	S. Disagree
Colostrum milk impairs growth and development	5 (1.7%)	53 (17.6%)	31 (10.3%)	181 (60.1%)	31 (10.3%)
Colostrum causes diarrhea in an infant	4 (1.3%)	71 (23.6%)	32 (10.6%)	174 (57.8%)	20 (6.6%)
Colostrum is forbidden in culture	1 (03%)	69 (22.9%)	32 (10.6%)	185 (61.5%)	14 (4.7%)
Colostrum is a dirty part of milk	2 (0.7%)	67 (22.3%)	33 (11.0%)	183 (60.8%)	16 (5.3%)
Colostrum milk is difficult to digest and needs to be discarded	3 (1.0%)	61 (20.3%)	35 (11.6%)	186 (61.8%)	16 (5.3%)

### The practice of colostrum feeding

The proportion of colostrum feeding practice among mothers who attend postnatal care was found to be 57.8% (95% CI: 52.2–63.35) as indicated in Figure 1.

### Factors associated with colostrum feeding practice

The study employed both Bivariable and multivariable binary logistic regression analyses to identify the factors associated with colostrum feeding practice. In the bivariable analysis, factors with a *p*-value of less than 0.25 were maternal resident, the number under-five children, sex of neonate, ANC visiting, parity, place of birth, counseling on BF, and colostrum and maternal attitude. However, in the multivariable logistic regression analysis urban resident, health counseling on colostrum feeding, and good maternal attitude variables were found to be significantly associated with colostrum feeding at a *p*-value of < 0.05 (Table 6).

In multivariable logistic regression, the odds of practicing colostrum feeding were 4.05 times higher among mothers residing in urban areas compared to those living in rural areas (AOR = 4.05, 95% CI: 2.18–7.52). Mothers who received counseling on breastfeeding and colostrum had 4.3 times the odds of the practice of colostrum feeding compared to those who did not receive counseling (AOR = 4.31, 95% CI: 1.27–10.73). Those mothers with a favorable attitude toward colostrum milk had 6.6 times higher odds of colostrum feeding practice than mothers with an unfavorable attitude (AOR = 6.64, 95% CI: 3.61–12.22) (Table 6).

**Table 6:** Binary and multivariable logistic regression analysis of factors associated with colostrum feeding practice among mothers who attended the PNC clinic at Asella referral and teaching hospital, Oromia regional state, Southeast Ethiopia, 2024 (*N* = 301).

Variable	Category	Colostrum feeding practice		COR (CI = 95%)	AOR (CI = 95%)
		Good	Poor		
Maternal residence	Urban	87 (50.0%)	27 (21.3%)	3.7 (2.08–6.73)	4.05 (2.18–7.51)**
	Rural	87 (50.0%)	100 (78.7%)	1	**1**
The number of children	<2	52 (40.9%)	75 (59.1%)	1	1
	≥2	90 (51.7%)	84 (48.3%)	1.55 (1.12–2.45)	1.73 (0.96–3.09)
Sex of neonate	Male	77 (44.3%)	72 (56.7%)	**1**	1
	Female	97 (55.7%)	55 (43.3%)	1.65 (1.04–2.61)	1.54 (0.86–2.73)
Monthly income	<5,000	34 (19.5%)	40 (31.5%)	1	1
	5,000–10,000	99 (56.9%)	61 (48.0%)	1.91 (1.09–3.33)	1.42 (0.68–2.91)
	>10,000	41 (23.6%)	26 (20.5%)	1.86 (0.95–3.63)	1.09 (0.46–2.57)
ANC visiting	Yes	169 (97.1%)	114 (89.8%)	3.85 (1.34–11.1)	5.01 (0.41–60.51)
	No	5 (2.9%)	13 (10.2%)	1	1
Counseling on BF and colostrum	Yes	39 (23.1%)	8 (6.9%)	4.05 (1.82–9.04)	4.31 (1.73–10.73)*
	No	130 (76.9%)	108 (93.1%)	1	**1**
Parity	Primiparity	58 (33.3%)	54 (42.5%)	1	**1**
	Multiparty	116 (66.7%)	73 (57.5%)	1.48 (0.92–2.37)	1.27 (0.70–2.31)
Place of birth	Home	6 (1.1%)	8 (3.9%)	1	1
	Health facility	168 (98.9%)	119 (96.1%)	1.9 (1.12–18.47)	3.01 (0.31–29.14)
Maternal attitude	unfavorable	33 (19.0%)	79 (62.2%)	1	1
	Favorable	141 (81.0%)	48 (37.8%)	7.03 (4.17–11.8)	6.64 (3.61–12.2)**

NB: In multivariable, the variables that have *p*-value < 0.05 have a truly significant association with colostrum feeding practice, CI: confidence interval, *p-value < 0.05, **p-value ≤ 0.001, COR, Crude odd ratio; AOR, Adjusted odd ratio. The bold value indicates significant association to outcome variable. p*<0.05, p**<0.005.

## Discussion

**Figure 3:**
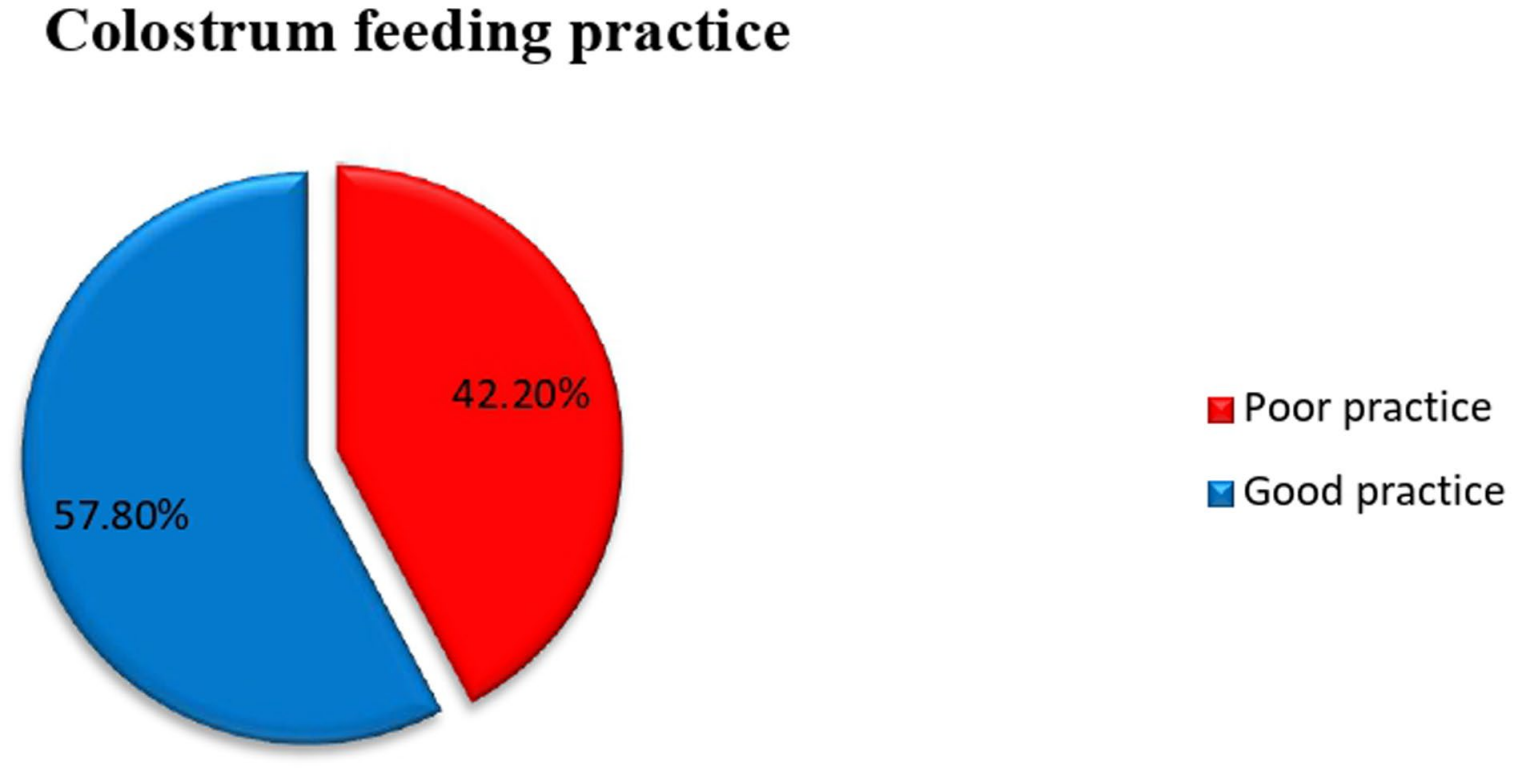
Prevalence of colostrum feeding practice among Mothers Who Come for Postnatal Care to Asella Referral and Teaching Hospital, Arsi Zone, South-East Ethiopia, 2024.

This study examined colostrum feeding practices and associated factors among postnatal mothers at Asella Referral and Teaching Hospital. The prevalence of colostrum feeding in the study area was found to be 57.8%. This result aligns with findings from studies conducted in Debre Berhan town (56.2%) (27) and Ambo (56.5%) (26). However, the prevalence observed in this study is lower compared to findings from other locations, such as the three districts in Sindh province, Pakistan namely, Larkana, Qamber Shahdadkot, and Dadu (72.1%) (19), Pakistan’s Military Hospital Rawalpindi (86%) (7), Papua New Guinea (68.6%) (28), Kamrul, Assam in India (76%) (29), Bahir Dar city (83.3%) (30), Arba Minch Zuria (89.0%) (31), Mizan Tepi University Teaching Hospital, Bench Maji Zone, SNNPR (60.88%) (32), Gununo Health Center in the Wolaita Zone (83%) (1), Dubti town, Afar regional state (84.4%) (21), Motta town (79.8%) (24), Hula district of Sidama Region (96.1%) (33), and Samara-Logia city, Northeastern Ethiopia (88.0%) (34). This variation could be attributed to differences in maternal access to healthcare services and the sociocultural practices surrounding breastfeeding (Figure 3).

The findings of this study were higher than those of a study conducted in Egypt, which reported a colostrum feeding rate of 41.4% (35). This difference may be attributed to variations in the study area and socio-demographic factors. Additionally, cultural beliefs and practices related to breastfeeding, including colostrum feeding, can differ significantly across regions and countries. Cultural norms, traditions, and taboos around infant feeding practices can strongly influence maternal behaviors and attitudes toward colostrum feeding. For example, in some cultures, colostrum may be viewed as harmful or unnecessary, resulting in lower rates of colostrum feeding.

This study also identified several factors associated with maternal colostrum feeding practices, including participant residence, counseling on the timely initiation of breastfeeding during antenatal care, and maternal attitudes toward colostrum feeding. The findings indicated that mothers residing in urban areas were more likely to practice colostrum feeding compared to those living in rural areas. This result is consistent with studies conducted in Kombolcha, South Wollo Zone of Ethiopia (29), governmental health facilities in Dire Dawa administrative city (8), and Kamrup, Assam in India, which also showed that urban mothers were more likely to provide colostrum than their rural counterparts (36). The possible explanation for this finding is the difference in access to healthcare services and education between urban and rural areas. Urban areas generally have better access to healthcare facilities, including hospitals and clinics, where mothers may receive more comprehensive antenatal care, including counseling on the importance of colostrum feeding. In contrast, rural areas may experience challenges such as limited access to healthcare services, lower levels of education, and traditional beliefs that may discourage colostrum feeding. These factors likely contribute to the observed disparity in colostrum feeding practices between urban and rural mothers in the study.

The finding of this study indicated that mothers who received health education counseling during antenatal care follow-ups regarding colostrum feeding were more likely to practice colostrum feeding compared to those who did not receive counseling. This finding is consistent with the study conducted in Wolaita Sodo (17). This could be because health education counseling during antenatal care (ANC) follow-ups provides mothers with information about the importance and benefits of colostrum feeding. This counseling may cover topics such as the nutritional value of colostrum, its role in protecting newborns from infections, and its contribution to early bonding between mother and child. When mothers receive this education, they are more likely to understand the significance of colostrum feeding and feel empowered to initiate and continue the practice after childbirth.

Mothers with a favorable attitude toward colostrum milk were more likely to have higher odds of colostrum feeding practice compared to mothers with an unfavorable attitude. The result is consistent with the study conducted in Addis Ababa (9). This might be due to a positive attitude toward colostrum lead to a stronger intention to practice colostrum feeding. Essentially, when mothers have a favorable attitude toward colostrum, they are more likely to perceive it as valuable and important, which translates into a higher motivation to engage in the behavior of colostrum feeding.

## Conclusion and recommendation

### Conclusion

The study area had a low rate of colostrum feeding practice (57.8%) when compared with WHO recommendations. Additionally, this study identified factors associated with maternal colostrum feeding practice level such as participant residence, counseling on timely initiation of breastfeeding during antenatal care, and maternal attitude toward colostrum feeding. Therefore, the following recommendation was forwarded to different stakeholders;

### Recommendations

**Health care professionals**

- Should conduct door-to-door campaigns in rural areas to educate expectant mothers about the importance of colostrum feeding.
- Provide Health education dissemination to all postnatal mothers on the importance of colostrum feeding to increase mothers’ attitude toward breast feeding.
- Should incorporate counseling on timely initiation of breastfeeding, including colostrum feeding, into their antenatal care consultations.

**Community Leaders and Volunteers**:

- Should work in collaboration with health care professionals and facilitate mobilization of rural community to enhance Health education on colostrum feeding to improve mothers Attitude.

**Future researchers**

- Consider conducting longitudinal studies to track maternal colostrum feeding practices over time. Longitudinal data can provide insights into changes in feeding behaviors and attitudes throughout pregnancy, childbirth, and the postpartum period, allowing for a more comprehensive understanding of the factors influencing colostrum feeding.

### Limitations of the study

The study is only conducted at Asella Referral and Teaching Hospital, which may limit the generalizability of the findings to other healthcare settings with different patient populations and practices. Additionally, the study is cross-sectional design which does not allow for the establishment of causal relationships. Moreover, there was recall bias due to study participants being unable to remember what happened in the past.

## Data Availability

The original contributions presented in the study are included in the article/supplementary material, further inquiries can be directed to the corresponding author.
