# Supplementary material for: Colostrum feeding practice and associated factors among mothers who come for postnatal care to Asella referral and teaching hospital, Arsi Zone, South-East Ethiopia
Source: Front Med (Lausanne). 2025 Jan 7;11:1487179. doi: 10.3389/fmed.2024.1487179 (PMC11747558; doi:10.3389/fmed.2024.1487179)
Supplement: Supplementary file 1 [file Table_1.DOCX]

**English version Questionnaire**

**Instructions:** There are a series of questions to be responded by participants.

**Direction**: - please encircle the letter of the participant's answer choice or correctly fills in the blank space provided.

Code number of the participant____________________

| S.NO | | Question | Response | Remark | |  |
| --- | --- | --- | --- | --- | --- | --- |
| 1. **Household demographic and socio-economic data** | | | | | |  |
| 101. | | Age of mother | ________________yrs. |  | |  |
| 102. | | Resident | 1. Urban 2. Rural |  | |  |
| 103. | | Religion | 1. Orthodox  2. Muslim  3. catholic  4. protestant  5. Others (specify)………. |  | |  |
| 104. | | What is your ethnicity? | 1. Amhara  2. Oromo  3. Tigre  4. Gurage  5.Others (specify)__________ |  | |  |
| 105. | | Current marital status of a mother | 1. Unmarried 2. Married 3. Divorced 4. Widowed |  | |  |
| 106. | | Education status of a mother | 1. Can’t read and write  2. Read and write  3.Primary school(1-8)  4.Secondary school(9-10)  5. College and above |  | |  |
| 107. | | Occupation of mother | 1. Housewife  2. Government employee  3. Daily laborer  4. Merchant  5. Other (specify)……….. |  | |  |
| 108. | | Educational status of the father | 1. Can’t read and write 2. Read and write 3. Primary school(1-8) 4. Secondary school(9-10) 5. College and above |  | |  |
| 109. | | Occupation of father | 1. Government employee  2. Farmer  3. Daily laborer  4. Merchant  5. Others (specify)……………… |  | |  |
| 110. | | How many children under five do you have? | _______________children |  | |  |
| 111. | | Total family size | _______________ |  | |  |
| 112. | | Estimated average monthly household income | ___________________Birr |  | |  |
| 113. | Sex of the child | | 1. Male 2. Female |  | |  |
| 114. | | Has the child growth monitoring been followed up? | 1. No 2. Yes |  | |  |
| 1. **Health service-related characteristics** | | | | | |  |
| 301. | | Did you receive antenatal care for your last pregnancy? | 1. No 2. Yes | If 1 skip to  304 | |  |
| 302. | If yes for Q No.301. How many times did you receive ANC? | | ____________________times. |  | |  |
| 303. | Did you receive Health education on IYCF practice during your ANC visits? | | 1. No 2. Yes |  | |  |
| 304. | Where did the child born? | | 1. Home 2. Health facility   99.Other, specify____________ |  | |  |
| 305. | Did you receive PNC care for your last delivery? | | 1. No 2. Yes | If 1 skip to  308 | |  |
| 306. | If yes for Q No.305. How many times did you receive PNC? | | ____________________times. |  | |  |
| 307. | Did you receive Health education on IYCF practice during your PNC visits? | | 1. No 2. Yes |  | |  |
| 308. | Has your child been vaccinated? | | 1. No 2. Yes |  | |  |
| **ІV. Child feeding Practice** | | | | | |  |
| 401. | Did you provide colostrum to the baby after birth? | | 1. No 2. Yes | | If 1 skip to  403 |  |
| 402. | If not why? | | ____________________ specify. | |  |  |
|  | When did you provide colostrum to the baby after birth? | | 1. Within 1 hour after delivery | |  |  |
|  |  |  | 2. Within 6 hour after delivery | |  |  |
|  |  |  | 3. Within 24 hour after delivery | |  |  |
|  |  |  | 4. After discard some of colostrum milk | |  |  |
| 403 | Did you provide pre lacteal feeding to your baby? | | 1. No 2. Yes | | If 1 skip to 501 |  |
| 404. | What did you give (feed) your baby within the first three days after delivery, before your white milk began flowing regularly? | | 1. Cow’s milk. 2. Honey 3. Butter. 4. Water and sugar 5. Colostrum 6. Nothing fed. 7. Others___________ specify | |  |  |
| \| 1. **Knowledge about colostrum feeding** \| \| \| \| --- \| --- \| --- \| \| **No** \| **Questions** \| **Response** \| \| Q501 \| Did you know colostrum milk? \| 1. No 2. Yes \| \| Q502 \| Colostrum is thick, sticky, and yellowish \| 1. No 2. Yes \| \| Q503 \| If you know from where did you hear? \| 1. No 2. Yes \| \| Q504 \| Colostrum feeding is initiated within an hour and continue to three days after birth \| 1. No 2. Yes \| \| Q505 \| Colostrum is high in protein & helps the baby to grow \| 1. No 2. Yes \| \| Q506 \| Colostrum is first vaccine given to the baby \| 1. No 2. Yes \| \| Q507 \| Colostrum is first vaccine given to the baby \| 1. No 2. Yes \| \| Q508 \| Colostrum milk protects vaginal bleeding? \| 1. No 2. Yes \| \|  \| \| \| | | | | | | |

1. **Attitude towards colostrum feeding practice**

| **1-Strongly disagree , 2-Disagree , 3-Neutral , 4-Agree, and 5-Strongly Agree** | | | | | |
| --- | --- | --- | --- | --- | --- |
| Colostrum milk impairs growth and development | Strongly disagree | Disagree | Neutral | Agree | Strongly agree |
| Colostrum causes diarrhea for an infant | 1 🗖 | 2🗖 | 3🗖 | 4🗖 | 5🗖 |
| Colostrum is forbidden in culture | 1🗖 | 2🗖 | 3🗖 | 4🗖 | 5🗖 |
| Colostrum is a dirty part of milk | 1🗖 | 2🗖 | 3🗖 | 4🗖 | 5🗖 |
| Colostrum milk is difficult to digest & needs to be discard | 1🗖 | 2🗖 | 3🗖 | 4🗖 | 5🗖 |

**Thank you for your co-operation**
